# Integrative analysis reveals novel driver genes and molecular subclasses of hepatocellular carcinoma

**DOI:** 10.18632/aging.104047

**Published:** 2020-11-20

**Authors:** Liguang Yang, Zhengtao Zhang, Yidi Sun, Shichao Pang, Qianlan Yao, Ping Lin, Jinming Cheng, Jia Li, Guohui Ding, Lijian Hui, Yixue Li, Hong Li

**Affiliations:** 1 Bio-Med Big Data Center, CAS Key Laboratory of Computational Biology, CAS-MPG Partner Institute for Computational Biology, Shanghai Institute of Nutrition and Health, Shanghai Institutes for Biological Sciences, Chinese Academy of Sciences, Shanghai 200031, China; 2 University of Chinese Academy of Sciences, Beijing 100049, China; 3 State Key Laboratory of Cell Biology, CAS Center for Excellence in Molecular Cell Science, Shanghai Institute of Biochemistry and Cell Biology, Chinese Academy of Sciences, Shanghai 200031, China; 4 School of Life Sciences and Biotechnology, Shanghai Jiao Tong University, Shanghai 200240, China; 5 Department of Pathology, Fudan University Shanghai Cancer Center, Shanghai 200032, China; 6 Department of Oncology, Shanghai Medical College, Fudan University, Shanghai 200032, China; 7 Anhui Engineering Laboratory for Big Data of Precision Medicine, Anhui 234000, China; 8 Collaborative Innovation Center of Genetics and Development, Fudan University, Shanghai 200433, China; 9 Shanghai Center for Bioinformation Technology, Shanghai Academy of Science and Technology, Shanghai 201203, China

**Keywords:** systematic integration, epigenome, stratifin, SPP1, liver cancer

## Abstract

Hepatocellular carcinoma (HCC) is a heterogeneous disease with various genetic and epigenetic abnormalities. Previous studies of HCC driver genes were primarily based on frequency of mutations and copy number alterations. Here, we performed an integrative analysis of genomic and epigenomic data from 377 HCC patients to identify driver genes that regulate gene expression in HCC. This integrative approach has significant advantages over single-platform analyses for identifying cancer drivers. Using this approach, HCC tissues were divided into four subgroups, based on expression of the transcription factor E2F and the mutation status of TP53. HCC tissues with E2F overexpression and TP53 mutation had the highest cell cycle activity, indicating a synergistic effect of E2F and TP53. We found that overexpression of the identified driver genes, stratifin (SFN) and SPP1, correlates with tumor grade and poor survival in HCC and promotes HCC cell proliferation. These findings indicate SFN and SPP1 function as oncogenes in HCC and highlight the important role of enhancers in the regulation of gene expression in HCC.

## INTRODUCTION

Hepatocellular carcinoma (HCC) is the second leading cause of cancer-related mortality worldwide [1]. In the United States, the incidence of HCC has been rising faster than any other cancer type [2]. Common risk factors associated with HCC include hepatitis B or C infection, high alcohol consumption, autoimmune hepatitis, and metabolic diseases [3]. The intra-tumor and interpatient heterogeneities in HCC [4–6] make early detection and effective therapies difficult, leading to the low five-year survival rates [7, 8]. Thus, it is crucial to understand the molecular mechanisms driving HCC.

Genome sequencing of HCC cohorts has revealed many genomic alterations, such as mutations of TP53, CTNNB1, and AXIN1, copy number alterations of CDKN2A/CDKN2B, and multiple alterations of TERT [9–14]. These genes are called drivers to distinguish them from randomly occurring passenger alterations. In addition to genetic drivers, epigenetic drivers may also play important roles in HCC [15–18]. While global DNA hypomethylation makes the genome unstable, promoter hypermethylation may silence tumor suppressor genes, such as SMPD3 and NEFH [19]. Since drivers contribute to tumorigenesis by different mechanisms, there is a need to identify the HCC drivers by integrative analysis of multiple omics data.

Current progress in computational algorithms demonstrates that integrative analysis of multi-omics data could increase our ability to identify likely drivers. For example, CONEXIC integrated matched copy number and gene expression data to identify the combination of modulators in melanoma [20]. Lemon-tree reconstructed module network and identified upstream regulators from multi-omics datasets [21]. Additionally, integrated analysis of multi-omics data may resolve cancer molecular classification with clinical relevance, and reveal previously unrecognized subgroups. The Cancer Genome Atlas (TCGA) study performed the first large scale multi-platform analysis of HCC [14]. They identified oncogenic drivers with significant mutations or copy number alterations, and found three subtypes by multi-platform integrative clustering. However, their integration approach did not consider the function effects of drivers. The shared data of TCGA project provide a valuable resource for us to improve the integration analysis. Since gene expression is an intermediate molecular phenotype that connects genome and phenotype, we hypothesize that alterations that regulate the expression of multiple genes are more likely to be the real drivers.

In this study, we performed an integrative analysis using five platform data from 377 HCC patients to identify driver genes that regulate gene expression in HCC. The integrative approach has obvious advantages over single-omics analysis. In addition, we utilized the driver events to classify HCC tissues into four subclasses having distinct prognostic implications and molecular characteristics. Furthermore, we used histone modification data to deepen our understanding of the regulation of gene expression in HCC.

## RESULTS

### Identification of HCC drivers by multi-omics integration

**Figure 1:**
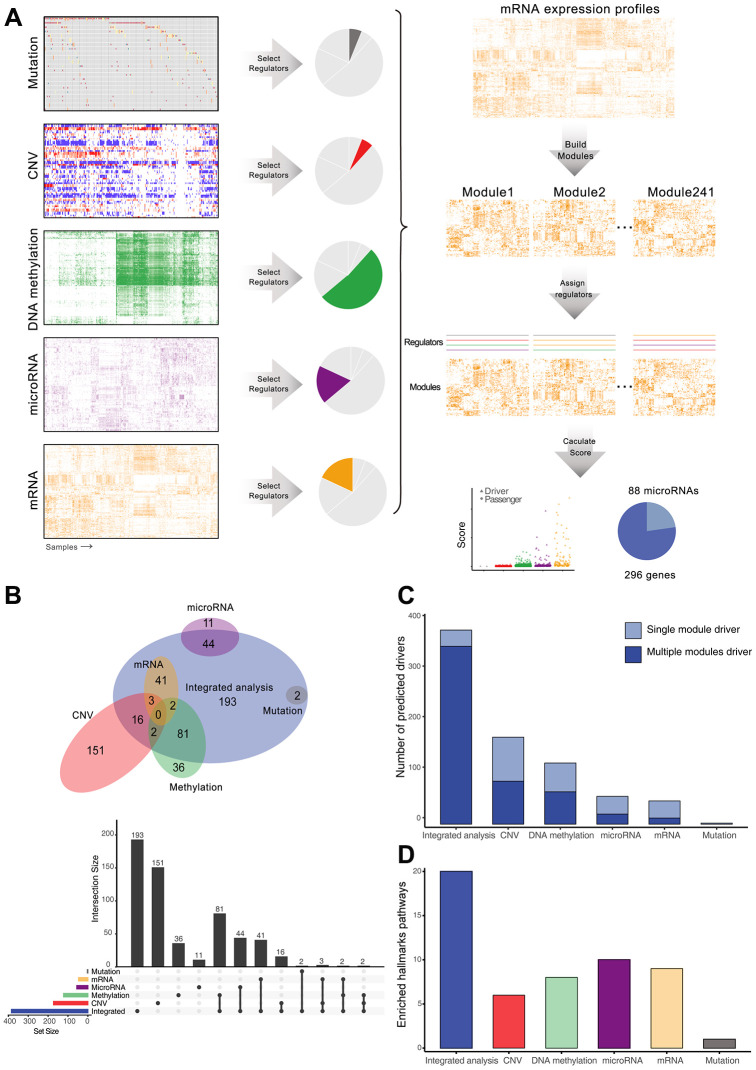
**Identification and comparison of HCC drivers by integrative and single-platform approach.** (**A**) Overview of the computational scheme for the systematic identification of drivers in HCC patients. Gray, red, green, purple, and orange represent mutations, copy number, DNA methylation, microRNA and mRNA, respectively. (**B**) Venn diagram and upset plot showing drivers recognized by different methods. (**C**) Regulation ability of drivers, measured by the number of modules. (**D**) Number of significantly enriched cancer hallmarks of driver genes.

Multiple-omics data of 377 HCC tissues and 50 adjacent normal tissues were collected from TCGA project, including somatic mutations, copy number alterations, DNA methylation, and mRNA and miRNA expression. 9560 genes differentially expressed between cancer and normal tissues were divided into 241 co-expression modules by Gibbs sampling cluster algorithm. Potential upstream regulators were obtained from single-platform analysis or literature, including 69 mutated genes, 886 amplified and 1829 deleted genes, 570 differentially methylated genes, 196 differentially expressed microRNAs, 121 transcription factors, and 76 HCC associated genes (Supplementary Table 1, Materials and Methods) These candidate regulators were assessed by an integrative multi-omics module network inference algorithm [21], which inferred the regulation relationship between co-expression modules and their associated regulators. High-scoring regulators were chosen as cancer drivers that might drive development of HCC. The final driver list was composed of 296 protein-coding genes and 88 microRNAs, of which 166 genes were not reported previously (Figure 1A, Supplementary Table 2, Supplementary Figure 1A).

To compare the differences between multi-omics and single-omics analyses, we utilized the same process, but only assigned candidate regulators from each single platform to identify the drivers. Only two significant drivers (TP53 and CTNNB1) regulating expression modules were identified from mutation data because of the low mutation frequency, but 172, 121, 55 and 46 drivers were identified from CNV, methylation, miRNA, and mRNA datasets, respectively. The multi-omics integrated analysis covered most of the drivers identified from single-omics analysis, but also identified new drivers (Figure 1B). Moreover, multi-omics integration identified drivers that simultaneously regulated multiple modules (Wilcoxon rank sum test *p* = 0.0054, Figure 1C). To investigate the association between driver genes and cancer hallmarks, we performed gene set enrichment analysis for drivers and found that the drivers generated from integrated analysis were significantly enriched in cancer hallmarks pathways (Chi-square test, *p* = 1.194 × 10^-5^ Figure 1D, Supplementary Figure 1B). These results indicate that the integrative approach can more accurately detect cancer drivers with functional implications.

### Functions and characteristics of HCC drivers

**Figure 2:**
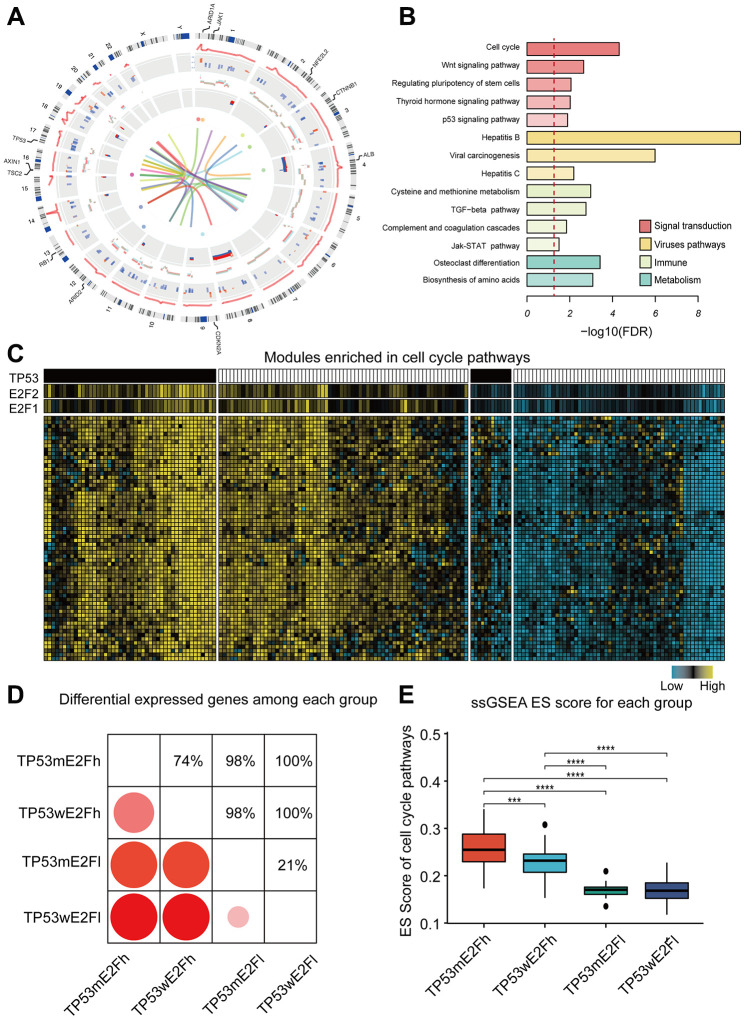
**Functions and characteristics of HCC drivers.** (**A**) Circos plot shows the alterations of 384 drivers. Circular tracks from outside to inside: regulation score, mRNA fold-change, copy number alteration, DNA methylation status, mutations, and protein interactions. The outermost labels indicate the significant mutated genes. Red represents high expression/methylation and CNV gain; blue represents low expression/methylation and CNV loss. Sex chromosomes are excluded. (**B**) Enriched KEGG pathways of the drivers; the dotted line indicates FDR=0.05. (**C**) Example of a co-expression module and its predicted regulators. Genes in this module are enriched in cell cycle pathway. HCC tissues were classified into four subgroups based on the combined alterations of E2F expression and TP53 mutation. (**D**) Comparison of the number of differentially expressed genes between any two subgroups. Only genes in the example module were used. (**E**) Comparison of the activity of cell cycle pathway among the four subgroups. ssGSEA was performed for each tumor using the genes in cell cycle pathway. Enrichment score (ES) represents the activity level of the pathway. TP53m and TP53w mean TP53 mutation and TP53 wildtype; E2Fh and E2Fl mean high and low expression of E2F. P value was determined by Wilcoxon rank sum test. ****P < 0.0001; ***P < 0.001; **P < 0.01;*P < 0.05.

Characteristics of the drivers generated from multi-omics integration are summarized in a circos plot (Figure 2A). Most of the drivers were down-regulated and hypermethylated in HCC tissues compared with normal tissues. Some drivers were enriched in copy number altered regions, such as chromosome 4q and 8q. We correctly identified known cancer genes, including 12 oncogenes, 42 tumor suppressor genes (TSGs), and 11 significantly mutated genes. We also found a number of novel driver genes that were not previously associated with HCC, including ANXA13, G6PC, STAT1, and JAK1. Some of them were significantly altered in at least two types of omics data, or were reported to participate in progression of other cancers [22–25].

Next, we performed a functional enrichment analysis to explore the biological function of these drivers. Tox analysis of these drivers showed that the most significantly associated toxicity phenotypes and clinical pathology endpoints included hepatocellular carcinoma, liver hyperplasia, and liver cirrhosis, corroborating our analysis (Supplementary Figure 2A). KEGG pathway analysis showed that the drivers were enriched not only in well-known pathways, such as cell cycle, WNT signaling, p53 signaling and HBV/HCV virus-related carcinogenesis, but also in several immune-related pathways, such as TGF-beta signaling, JAK-STAT signaling, and complement and coagulation cascades (Figure 2B). These pathways can be disrupted by different genes (Supplementary Figure 2B), and some genes have mutually exclusive mutation patterns (Supplementary Figure 2C, 2D); for example, CTNNB1, AXIN1 and APC in Wnt pathway, or RB1, CDKN1A and RBL1 in cell cycle pathway. Similar mutually exclusive patterns were observed in different alteration types of a single gene, such as a copy number deletion and mutation of AXIN1 (one-sided binomial tests *p* = 0.001). Such diverse alteration patterns may partly explain the heterogeneity of HCC.

Drivers have the potential to regulate expression of downstream genes. For example, genes in a 62-gene co-expression module were enriched in cell-cycle, and the predicted regulators were E2F1 expression, E2F2 expression, and TP53 mutation (Figure 2C). This is consistent with the existing knowledge that E2F transcription factor family and TP53 are crucial for cell cycle regulation. To explore the potential combination effect of E2F and TP53 on cell cycle, we classified HCC into four subgroups based on the expression of E2F1/E2F2 and the mutation status of TP53, and compared gene expression and cell cycle pathway scores among the four subgroups (Figure 2D–2E). Interestingly, HCC samples with E2F overexpression and TP53 mutation had the highest activity level of cell cycle pathway, indicating the synergistic effect of E2F and TP53.

### Regulatory network of HCC drivers and experimental validation of typic driver genes

**Figure 3:**
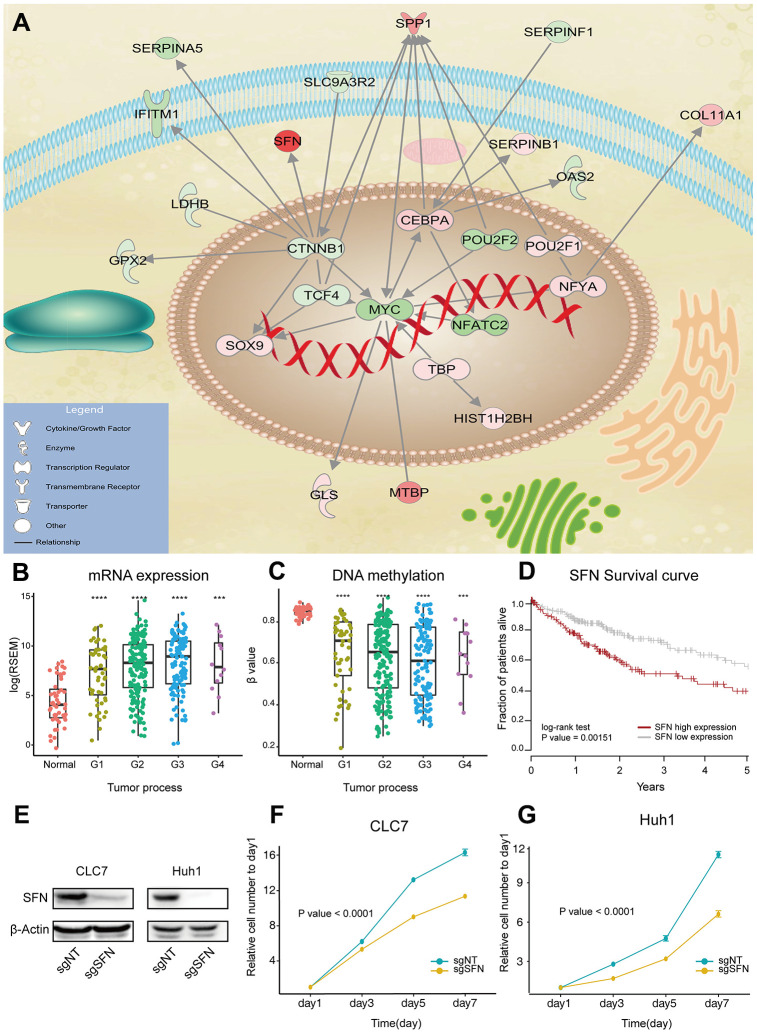
**SFN functions as oncogene in HCC.** (**A**) Max regulatory network of drivers, generated by IPA analysis. Edges represent direct regulatory relationships obtained from literature. Red and green nodes represent genes that are increased and decreased in HCC, respectively. (**B**, **C**) SFN expression and methylation correlate with HCC progression. P value was determined by Wilcoxon rank sum test: ****P < 0.0001; ***P < 0.001; **P < 0.01; **P < 0.01. (**D**) Survival of HCC patients with high and low SFN expression. (**E**) SFN protein levels analyzed by western blotting in SFN knockout cells. (**F**, **G**) Proliferation of CLC7 and Huh1 cells transfected with sgRNAs targeting SFN (n=3, regression analysis).

To understand connections between the identified drivers, we constructed literature-based regulatory networks using IPA. The functional categorization of the largest network revealed an important role in the regulation of migration and differentiation of tumor cells (Figure 3A). Some genes in this network have clear roles in HCC carcinogenesis, such as CTNNB1 and MYC. As for the rarely reported genes, we evaluated their expression changes in three independent datasets (GSE77314, GSE77509 and GSE97214), and analyzed their correlation with survival time and tumor grade. We found that the stratifin gene SFN was highly overexpressed in HCC (>8 fold in all datasets). Importantly, the increased expression of SFF and its decreased methylation correlated with the tumor grade (Figure 3B, 3C), and poor survival (Figure 3D).

SFN encodes 14-3-3 sigma protein, which regulates cell cycle and inhibits cysteine-type endopeptidase involved in apoptosis. To evaluate the role of SFN in HCC tumorigenesis, two independent sgRNAs were used to knockout SFN in two HCC cell lines (CLC7 and Huh1) by CRISPR/Cas9 system. Western blotting showed that SFN protein expression was efficiently suppressed in SFN knockout cell lines, indicating functional inactivation of SFN (Figure 3E). Compared to parental cell lines, the cell lines with suppressed SFN exhibited a markedly reduced proliferation (Figure 3F, 3G). Similar results can be observed with another highly expressed gene SPP1 (Supplementary Figure 3A–3D).

In order to further verify the reliability of the genes we obtained, we selected the top 50 genes with the highest integrative scores and upregulated in tumors for siRNA screening. Total 23 genes significantly inhibited cell proliferation after siRNA knockout, 17 of which were not reported in the literature (Supplementary Figure 3E). These experiments reveal the robustness of our results and overexpression of SFN and SPP1 in HCC promotes cancer cell proliferation.

### Multiple-platform determinants of HCC subclasses

**Figure 4:**
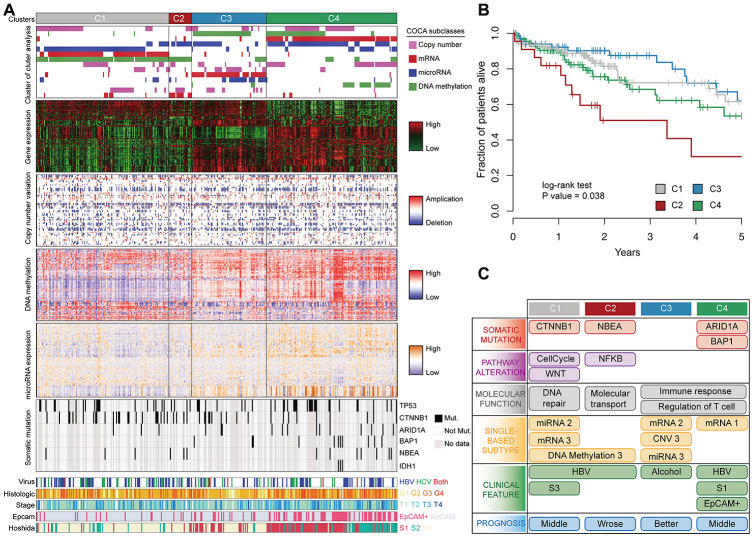
**Multi-omics integration to identify HCC subclasses.** (**A**) COCA subclasses of HCC identified by integrating multiple-platform data. Each column represents a patient; grey color in the spectrum of somatic mutation means patients without exome sequencing data. (**B**) Kaplan–Meier estimates of overall survival among different patient subclasses. (**C**) Schematic summary of molecular and clinical characteristics of the four HCC subclasses.

Previous cancer classification used highly variable genes to categorize patients into subclasses. The TCGA multi-platform classification used non-redundant copy number regions and the most variable CpG sites, mRNA, and miRNA for integrative clustering [14]. These approaches usually use a large number of genes, but the gene functions are not obvious. Since driver genes are master regulators of gene expression, we stratified HCC by using multi-platform data of driver genes. Samples were first clustered using each single. platform, and then a cluster of cluster analysis (COCA) was performed to determine the integrative clusters [26]. This analysis revealed four robust HCC subclasses: C1 (n=128), C2 (n=22), C3 (n=72), and C4 (n=126) (Figure 4A). The COCA-subclasses displayed a higher similarity with mRNA subclasses compared with other platforms (Supplementary Figure 4A), similar to a previous study in oligodendroglial tumors^21^. To compare our results with the classic HCC classification, we applied Hoshida’s [27] expression signatures to classify patients into three subclasses: S1 (n=112), S2 (n=74), and S3 (n=176). C1 predominantly consisted of Hoshida S3 tumors, whereas C4 predominantly consisted of Hoshida S1 tumors.

We observed significant survival differences among the subclasses identified by integrative analysis (Log-rank test *p* = 0.03). C2 tumors had significantly worse prognosis than other tumors, and C3 tumors had a better prognosis (Figure 4B). However, there was no significant difference in overall survival using Hoshida’s classification or single-platform classification in TCGA cohort (Supplementary Figure 4B–4D). To understand the characteristics of each subclass, we associated the subclasses with clinical, molecular, and signaling pathway features (Figure 4C). C1 tumors contained mutations in cell-cycle (*P* = 0.0039) and WNT (*P* = 0.0031) pathways, and in CTNNB1 gene (*P* = 0.0053). Differentially expressed genes between C1 and other tumors were enriched in DNA repair and viral carcinogenesis. C2 tumors were characterized by the mutations in NF-κB pathway (P=0.018) and NBEA (P=0.018). NF-κB links inflammation to cancer development and progression [28], and its overexpression in tumor tissues has been associated with a poor prognosis in different types of tumor [29]. Genes specifically expressed in C3 and C4 tumors were associated with immune response and T cell regulation. C4 tumors contained increased mutations in ARID1A (*P* = 0.0035), BAP1 (*P* = 0.0017) and IDH1 (*P* = 0.05) genes, were enriched in a signature of EpCAM positivity (Chi-square test *P* = 1.9 × 10^-13^, and overexpressed AFP (*P* = 0.018).

### Roles of enhancers in HCC

Despite substantial advantages of multi-omics integration, expression changes of some driver genes cannot be explained by existing data. For example, copy number gains of AHR and PBX1I in tumors are significantly lower than in controls (Supplementary Figure 5). To investigate the epigenetic mechanisms of gene regulation, we defined chromatin states based on the combination of multiple epigenetic markers from the ENCODE project [30], and then defined enhancer regions by H3K4me1 and H3K27ac signals (see Materials and Methods).

**Figure 5:**
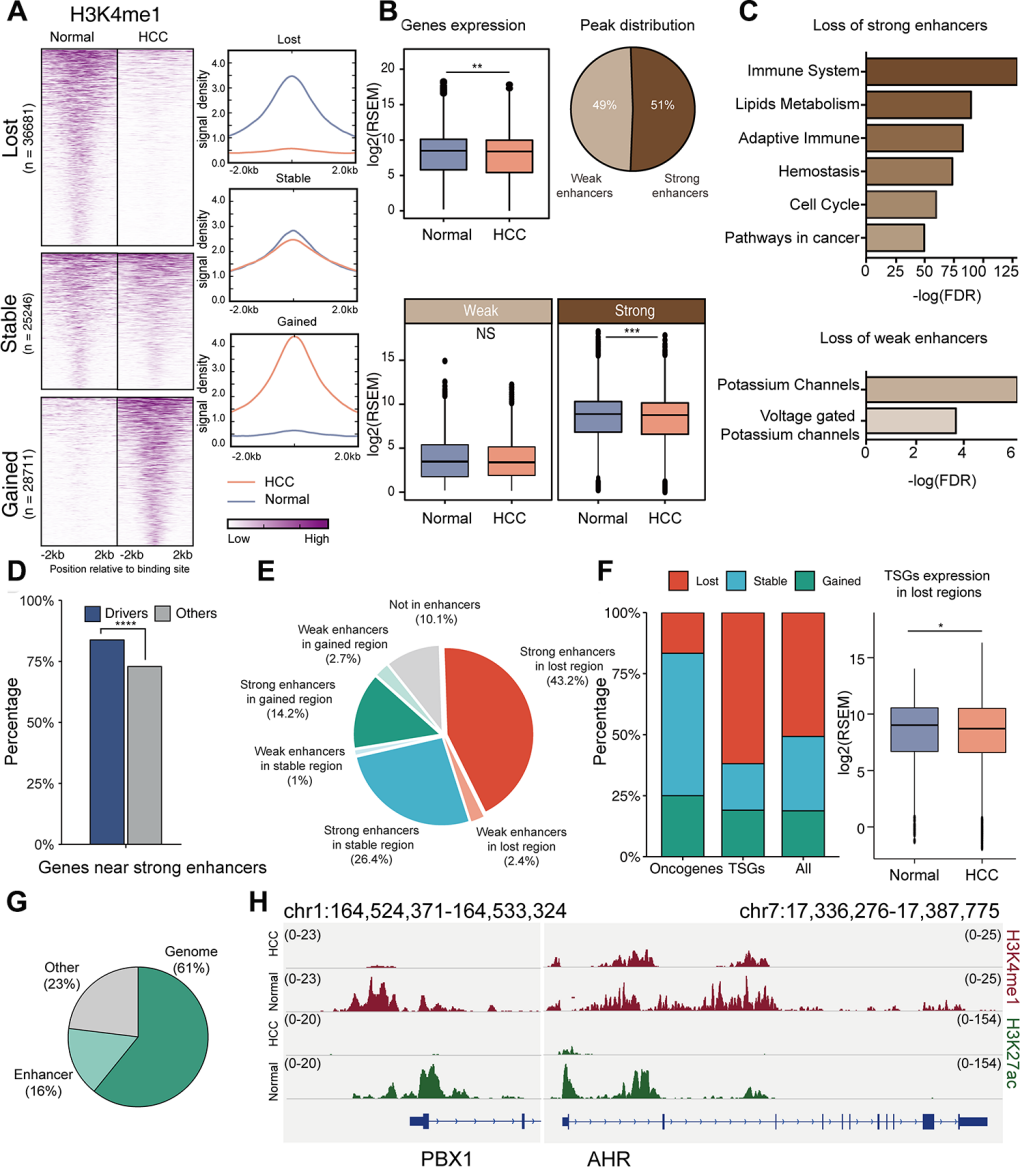
**Role of enhancers in HCC.** (**A**) Comparison of H3K4me1 signals in HCC cells (HepG2) and normal liver tissues. (**B**) Gene expression in enhancer loss regions in HCC and normal tissues. Enhancers were subdivided into strong and weak enhancers by H3K27ac signals. P value was determined by student’s t test: ***P < 0.001; **P < 0.01; *P < 0.05; NS:P ≥ 0.05. (**C**) Functional enrichment for genes associated with the inactivation of strong/weak enhancers. (**D**) Percentage of driver genes located in strong enhancers. P value was determined by chi-square test: ****P < 0.0001. (**E**) Distribution of drivers in different regions; the regions were divided according to the types of enhancers and their expression in HCC and normal tissues. (**F**) Comparison of enhancer alterations of oncogenes, TSGs and other drivers. (**G**) Proportion of drivers that change expression. “Genome” means proportion of drivers whose down-regulation may be caused by mutation, hyper-methylation, or copy number deletion; up-regulation may be caused by mutations or copy number amplification. “Enhancer” means proportion of drivers whose down-regulation may be caused by enhancer loss, and up-regulation may be caused by enhancer gain. (**H**) ChIP–seq signal tracks for H3K4me1 and H3K27ac in the regions around PBX1 and AHR.

Genes in actively transcribed regions exhibited increased expression compared to genes in inactive regions, and genes near strong enhancers exhibited increased expression compared to genes near weak enhancers (Supplementary Figure 6), reflecting the important regulatory role of chromatin states and enhancers [31]. Next, we compared the enhancer-related H3K4me1 signals between HCC and normal liver after excluding promoters, categorizing the genome into enhancer loss, enhancer stable, and enhancer gain based on the change in the H3K4me1 signal (Figure 5A, Supplementary Figure 7A). Loss of enhancer in HCC (40.8%) occurred more frequently than gain of enhancer (30.9%). This is consistent with a previous report demonstrating that the global enhancer expression is low in HCC [32]. We found that gene expression was significantly reduced in the regions with enhancer loss, especially those regulated by strong enhancers (Figure 5B). However, genes associated with stable enhancers and enhancer gain did not exhibit significant expression changes between HCC and normal tissues (Supplementary Figure 7B, 7C). We performed a functional enrichment analysis for genes associated with enhancer loss. In contrast to weak enhancers, inactivation of strong enhancers was associated with immunity and cancer pathways (Figure 5C), indicating that inactivation of strong enhancers plays an important role in HCC tumorigenesis.

Furthermore, we investigated the association between enhancer alterations and HCC driver genes. We first compared enhancers of driver genes with those of other genes. In normal liver, driver genes were located near strong enhancers compared to other genes (83.7% Vs. 72.9%, chi-square test, *p* = 7.37 × 10^−6^, Figure 5D). We also compared the enhancer alterations of driver genes between HCC and normal tissues, and found that the driver genes were associated with a loss of enhancers in HCC (45.6% loss vs. 16.9% gain, Figure 5E), similar with the low global expression of enhancers in HCC genomes. Next, we compared the enhancer alterations of well-known oncogenes, TSGs, and other drivers. TSGs were more likely to locate near the regions with enhancer loss (59.1%, chi-square test, *p* = 0.0793, Figure 5F), resulting in their decreased expression. Finally, we explored the role of enhancers in the regulation of driver gene expression. Copy number gain and promoter hypermethylation lead to increased expression, while somatic mutation was associated with both decreased and increased expression. Integrative analysis of copy numbers, mutations, and DNA methylation could explain the expression changes of 61% drivers, while enhancers could explain the expression changes of 16% drivers (Figure 5G). For example, although there was an obvious copy number gain in AHR and PBX1 genes, the lost enhancer signals might have resulted in their decreased expression in HCC (Figure 5H).

## DISCUSSION

The rapidly decreasing cost of omics experiments and increasing size of omics data have created an unprecedented opportunity to study cancer biology. However, multi-dimensional data pose a huge challenge for data analysis. In this study, we performed an integrative analysis of five platforms to identify driver genes and infer molecular classification of HCC. Compared to the original integrative analysis by TCGA network, our work is unique in several aspects: 1) Driver genes identified by TCGA are genes with significant somatic mutations or DNA copy number alterations; the TCGA approach does not consider the function effects of genomic alterations. Since we integrated several platforms and used a module network inference algorithm to evaluate the regulatory effects of genomic alterations on gene expression, the genes obtained by our approach are more likely to play important roles in HCC. 2) For multi-omics clustering, TCGA and our study used different genes and clustering algorithms. Since our study used driver genes instead of highly variable genes, the number of identified genes was smaller, and the subclasses had a better prognostic value than the TCGA subclasses. 3) Considering the alterations of histone methyltransferase [14], we analyzed histone modifications data and found the importance of gene enhancers for the regulation of gene expression.

Compared to single platform analysis, the integrative analysis of multi-platform data has obvious advantages. The integrative analysis can effectively remove random events from a single platform level and observe the true changes at multiple levels. The drivers identified from multi-omics analysis cover most of the drivers from single-omics, act predominantly as hub genes regulating multiple modules, and correlate well with cancer hallmarks. Multi-omics integrative analysis can also reveal the combined action of different omics leading to disorders of the same genes or pathways. Subgroups derived from integrative analysis have more obvious survival differences than results from single-platform analysis. Overall, integration of multi-omics data is an effective strategy for identifying key genes involved in carcinogenesis.

Our study provides a comprehensive list of candidate driver genes in HCC. It will be important to conduct follow-up experimental studies to validate the roles of the identified novel drivers in HCC. For example, our computational analysis showed that the increased expression of SFN and SPP1 were associated with higher tumor grades and worse survival outcomes, indicating that SFN and SPP1 might act as oncogenes in HCC. Indeed, our knockdown experiments demonstrated that SFN and SPP1 promoted migration of HCC cells, validating the computational analysis. Although the exact oncogenic mechanism of SFN and SPP1 remains to be elucidated, our data demonstrate that integrative analysis can discover novel drivers with biological importance. Identification of the mechanisms of these candidate drivers will provide a more comprehensive and systematic understanding of HCC.

Additionally, our analysis provides a unique insight into the role of enhancers in HCC. Previous studies reported that histone methyltransferase EZH_2_ was overexpressed in HCC, contributing to malignant transformation and poor prognosis [33, 34]. Due to the lack of histone modification data from a large HCC cohort, histone modifications cannot be directly integrated with other omics data. We used ChIP-seq data of H3K4me1 and H3K27ac obtained from HepG2 and normal liver tissues. We found that a loss of strong enhancers in HCC lead to a decreased expression of genes associated with carcinogenesis and immunity. Changes in the enhancer status could explain the expression changes of 16% drivers, which could not be explained by other types of omics data.

Together, our study shows that somatic mutations, copy number alterations, differential DNA methylation, and enhancer alterations coordinately regulate gene expression involved in hepatocarcinogenesis. A comprehensive driver list will provide a valuable resource for better understanding of the mechanisms responsible for HCC pathogenesis. The computational approach of integrating transcriptomic, genomic, and epigenetic data may be used also for other diseases.

## MATERIALS AND METHODS

### Data source

Multi-omics data of HCC were obtained from the Broad Institute TCGA Data Portal (http://firebrowse.org), including 377 HCC cases and 50 paired normal tissues on five platforms (RNA sequencing, DNA methylation arrays, miRNA sequencing, Affymetrix SNP arrays, and whole-exome sequencing). To minimize the possible batch effects, we applied the combat algorithm [35] to expression and methylation profiles.

### Identification of cancer driver genes by multi-omics integration

Genomic, epigenetic and transcriptomic data were used to select the candidate gene regulators (Supplementary Table 1). 1) Significantly mutated genes were identified by the Mutsig2CV algorithm. 2) Copy number amplified or deleted regions were obtained from GISTIC algorithms. 3) Differentially methylated genes were identified with MethylMix [36], which uses a beta mixture model to find differential and transcriptionally predictive methylation states. 4) Experimentally validated miRNA-target interactions were collected from miRTarbase Database [37]. Spearman correlation coefficients between microRNAs and target genes were calculated; significantly negatively correlated miRNA-targets were selected by FDR <0.05. A list of transcription factors (TFs) [38, 39] was obtained from the UCSC Genome Browser, and HCC associated genes were obtained from literature [9–13, 40, 41].

Differentially expressed genes between tumor and normal tissues (student t test with FDR < 0.05) were regarded as downstream genes. An integrative multi-omics module network was reconstructed by Lemon-Tree to predict causal regulators [21]. It first infers co-expression modules from expression profiles of downstream genes; Gibbs sampling procedure was used to update the cluster assignment of each gene and sample. Then it employs regulation programs by fuzzy decision trees, which use the candidate regulators to predict the mean expression of genes in a module. Finally, a regulator-to-module score was computed by regulatory programs for that module. Top 1% of high-scoring regulators for each module, or top 10% of high-scoring regulators for sum scores of all modules were regarded as potential cancer drivers [21].

### Functional enrichment analysis

Driver genes, genes in co-expression modules, and differentially expressed genes in HCC subclasses were subjected to functional enrichment analysis. Cancer hallmarks were downloaded from MSigDB [42]. Enriched biological pathways and disease processes were found by DAVID [43] and GSEA (FDR<0.05). Potential toxic effects and molecular networks were generated by Ingenuity Pathways Analysis software (IPA) [44].

### Cell culture

HCC cell line Huh1 was purchased from Japanese Collection of Research Bio-resources (JCRB, http://cellbank.nibiohn.go.jp/english/). HCC cell line CLC1 and CLC7 was established from Chinese HCC patients by Dr. Lijian Hui’ lab. CLC1 and CLC7 cells were cultured in RPMI 1640 (HyClone) supplemented with 10% FBS (HyClone), 1 × ITS (Insulin, Transferrin, Selenium Solution), 40 ng/mL EGF (epithelial growth factor), 100 U/ml penicillin, and 100 μg/ml streptomycin. Huh1 cells were cultured in DMEM (Gibco) supplemented with 10% FBS, 100 U/ml penicillin, and 100 μg/ml streptomycin. Both cell lines were maintained in a humidified incubator at 37°C with 5% CO2, and passaged every 3 days.

### SFN and SPP1 knockout

To knockout SFN and SPP1, we used CRISPR/Cas9 system. 20-nucleotide single-guide RNA (sgRNA) sequences targeting SFN (5’ AGCCTTCATGAAAGGCGCCG) and SPP1 (5’ TGTACTCACTGGTATGGCAC) were designed using the CRISPR design tool at http://crispr.mit.edu/. The sgRNA was cloned into the lentiCRISPR V2 vector (Addgene plasmid # 52961) and co-transfected with psPAX2 (Addgene plasmid # 12260) and pMD2.G (Addgene plasmid # 12259) packaging plasmids into 293FT cells. Virus containing supernatants were collected 48 h after transfection, filtered to remove cells using 0.45 um filter, and then used to infect HCC cell lines CLC1, CLC7 and Huh1 in the presence of 8 ug/ml polybrene. 16 h post-transfection, cells were selected with puromycin (1 ug/ml; 72 h); SFN silencing was confirmed by Sanger sequencing and western blotting.

### Cell proliferation assay

Cell proliferation was determined by CellTiter-Glo reagent (Promega). 50 μL of CellTiter-Glo reagent was added to each well of 96-well plates, and after a 10 min incubation at room temperature, luminescence was measured by EnVision Multilabel Reader (PerkinElmer), and normalized to day 1.

### SiRNA screening

A panel of 4X siRNAs for 50 genes (4 siRNAs/gene) were picked up from Human Genome siRNA library (Dharmacon) and dispensed 10 μl siRNA/well in 384-well plate by Biomek FX (Beckman Coulter). Three siRNAs were used as nontargeting control. 0.1 μl RNAimax (Invitrogen) in 10 μl serum-free opti-MEM medium (Thermo Fisher Scientific) was added by Multidrop Combi Reagent Dispenser (Thermo Fisher Scientific). Following a 20 min incubation, CLC7 cell line was seeded at a density of 3000 cells/well in 30 μl medium. The plate was transferred to the incubator for 96 hr. At the end point of treatment, each well was added 25 μl CellTiter-Glo reagent (Promega), and after 10 min incubation in room temperature, the luminescent signals were measured by EnVision Multilabel Reader (PerkinElmer) to determine the cell viabilities. The siRNA screening was performed twice.

### Multi-platform based molecular subclasses

Cluster of Cluster analysis (COCA) [26] was used to classify HCC into subclasses. First, using drivers as features, data from each molecular platform (copy number alteration, DNA methylation, mRNA, and microRNA expression) were clustered as described [26]. The robustness of the clusters was estimated according to the factorization rank and cophenetic correlation coefficient. Then, a second-level clustering was derived from the class assignments of individual molecular platforms, and the optimal combination of clusters was determined by maximizing the consensus cumulative distribution function. The final COCA subclasses represent a common cluster among the single platforms.

Using differentially expressed genes in each subclass, pathway enrichment analysis was done to assess the potential functions of the subclasses. Frequently mutated pathways in each subclass were evaluated by the proportion of tumors with any gene alteration. Survival curves were estimated and plotted using the Kaplan-Meier method. Log-rank tests were used to compare the survival curves among COCA subgroups. To associate molecular subclasses with clinical variables, Fisher’s exact test and two-way analysis of variance (ANOVA) were used to examine the difference in categorical and continuous variables among COCA subclasses, respectively. All statistical analyses were performed in R.

### ChIP-Seq analysis

ChIP-seq data of H3K4me1 and H3K27ac for HCC cell line (HepG2) and liver tissues were downloaded from the ENCODE database. ChIP-seq signal tracks were quantile-normalized using Haystack [45] with 50-bp windows. The blacklisted regions were downloaded from ENCODE and excluded in subsequent analysis. Promoter regions were identified by HOMER v4.10 [46]. The remaining differential peaks between HCC and normal tissues were determined by 2x fold-change. Heatmap and profile plot were generated using DeepTools v2.1.0 [47]. Genes associated with ChIP-seq peaks were identified via GREAT [48] with the basal plus extension (20kb) association rule. Genomic contexts were visualized by Integrative Genomics Viewer (IGV) software [49].

### Chromatin state and enhancer

To delineate the chromatin contexts, 18 chromatin states (E1~E18) of normal liver tissues and HCC cells (HepG2) were downloaded from http://compbio.mit.edu/roadmap [30]. Genome was subdivided into active regions (E1~E12) and inactive regions (E13~E18) by the chromatin state composition. H3K4me1 and H3K27ac are histone markers for active enhancers. Regions absent promoters with both H3K4me1 and H3K27ac marks were called strong enhancers, while regions with only H3K4me1 marks were called weak enhancers. Wilcoxon rank sum test was used to compare gene expression levels in different genomic regions. Chi-square test was used to compare the occurrence of super-enhancers in HCC and normal samples.

## Supplementary Material

Supplementary Figures

Supplementary Table 1

Supplementary Table 2
