# Supplementary material for: Integrative analysis reveals novel driver genes and molecular subclasses of hepatocellular carcinoma
Source: Aging (Albany NY). 2020 Nov 20;12(23):23849–71. doi: 10.18632/aging.104047 (PMC7762459; doi:10.18632/aging.104047)
Supplement: Supplementary Figures [file aging-12-104047-s001.pdf]

SUPPLEMENTARY FIGURES

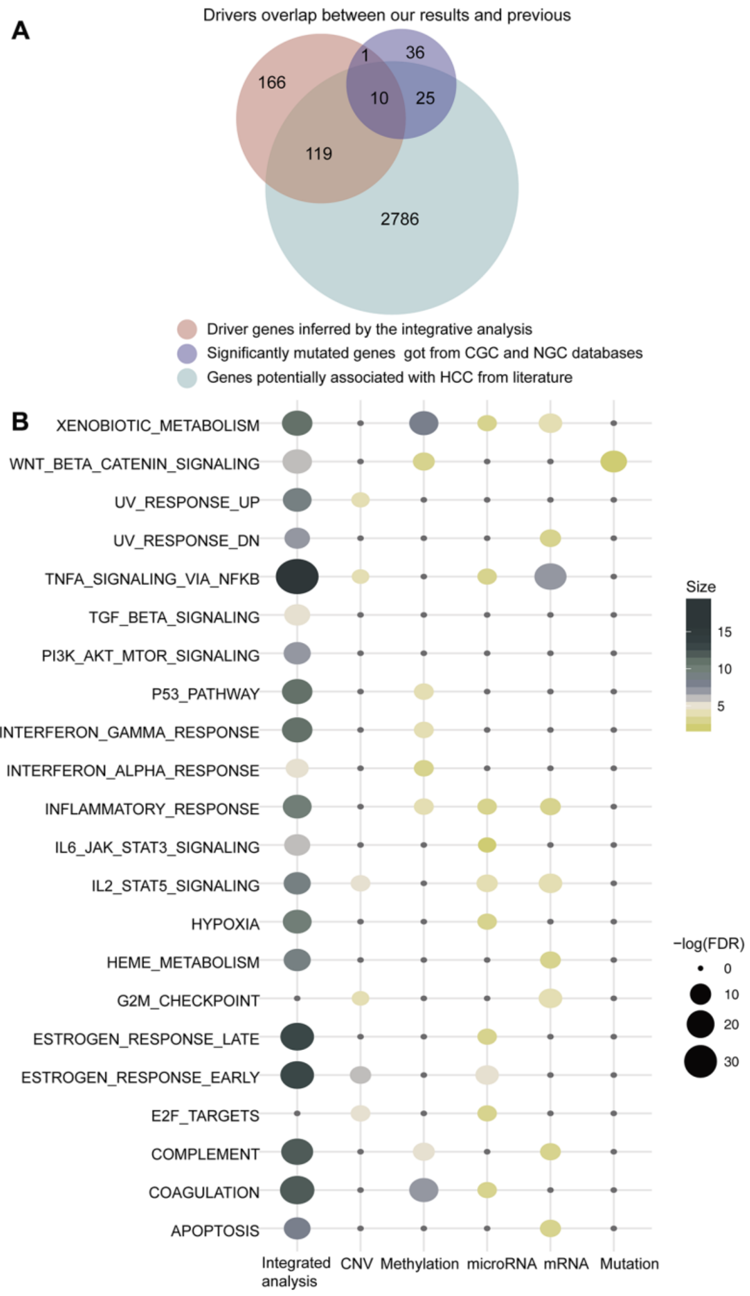

Supplementary Figure 1. Comparison between our results and previously published methods. (**A**) Comparison of the driver genes identified by our integrative analysis, and mutated genes and genes potentially associated with HCC identified from literature. Previously reported significantly mutated HCC-associated genes were obtained from Cancer Gene Census (CGC) and Network of Cancer Genes (NGC) databases. Genes potentially associated with HCC were obtained from PubMed by searching "hepatocellular carcinoma" and "gene" in the abstracts. (**B**) Cancer hallmarks enrichment for drivers identified by different omics data. Drivers from the integrated analysis were significantly enriched in the cancer hallmarks related terms, compared to independent analyses from each omics. Cancer hallmark terms were downloaded from MsigDB datasets. The size of the circle indicates the significance of the enrichment and the color indicates the number of drivers.

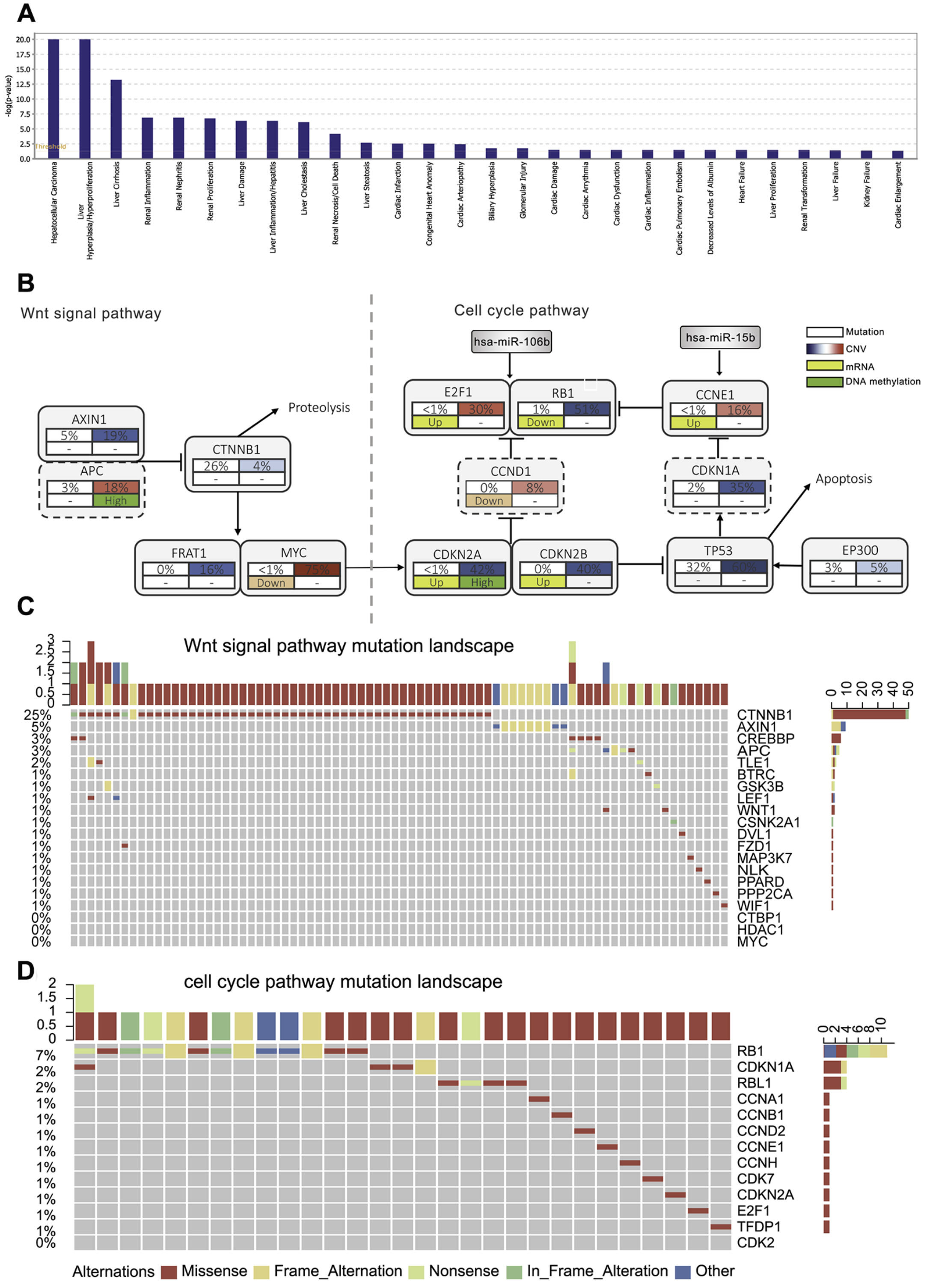

Supplementary Figure 2. Multi-omics landscape of Wnt and cell cycle pathways associated with HCC driver genes. (**A**) Tox analysis of drivers; hepatocellular carcinoma, liver hyperplasia/hyperproliferation and liver cirrhosis were significantly enriched, indicating correlation with liver cancer. (**B**) Overview of alterations in Wnt and cell cycle pathways. The solid rectangles represent drivers and dashed rectangles represent necessary linking genes in pathways that are not drivers. Colors represent the percentage of patients with mutations, copy number alterations, and dysregulation of methylation and expression. (**C**, **D**). Mutation landscape of Wnt and cell cycle pathway.

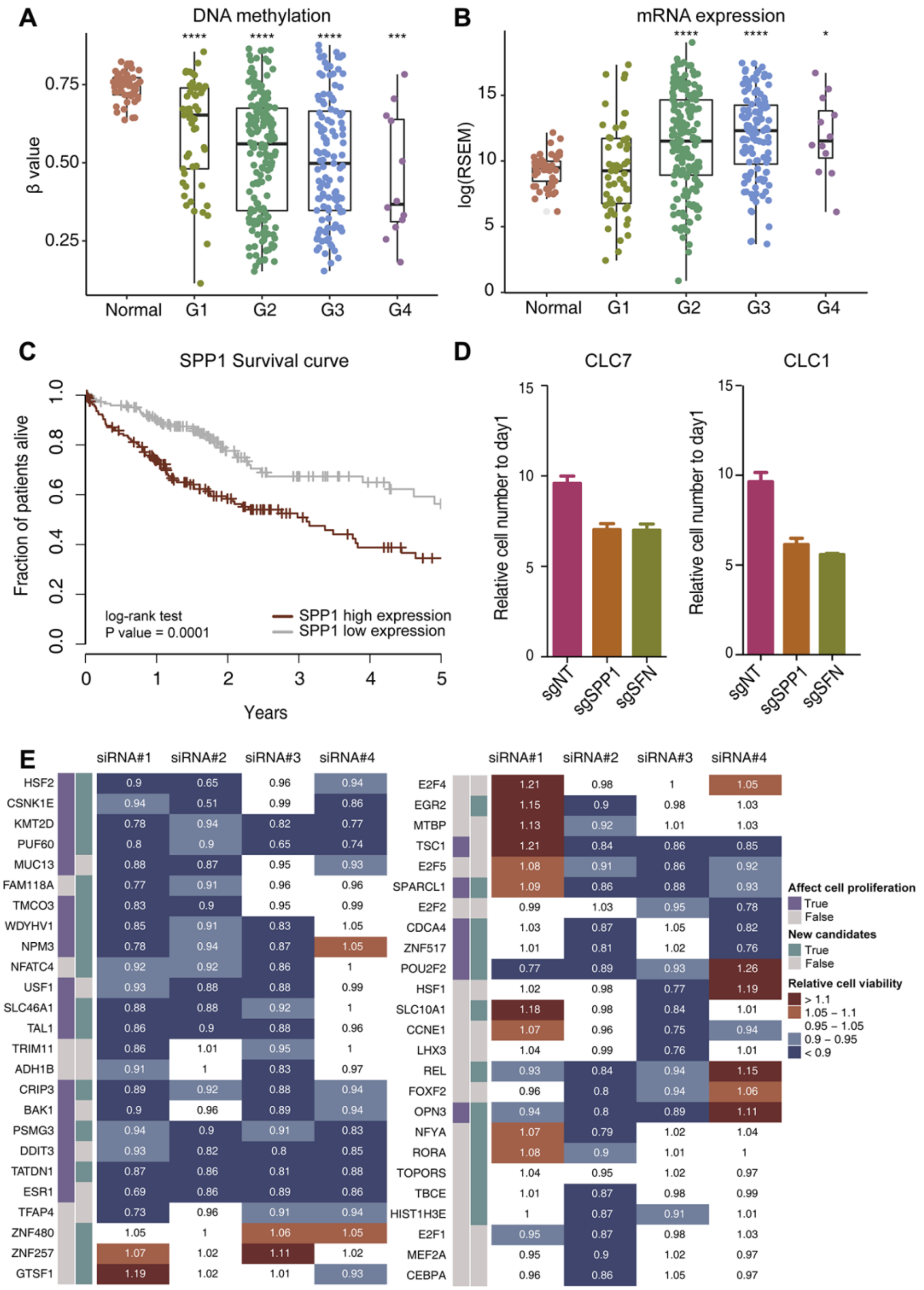

Supplementary Figure 3. Experimental validation of typic driver genes. (**A**, **B**) SPP1 methylation and expression correlate with HCC progression. P value was determined by Wilcoxon rank sum test: ****p <0.0001; ***p <0.001; **p < 0.01; *p < 0.05. (**C**) Survival of HCC patients with high and low SPP1 expression. (**D**) Proliferation of CLC7 and CLC1 cells transfected with sgRNAs targeting SPP1 and SFN (n=3, regression analysis) after 96 hours. The relative cell number is normalized by the first day. (**E**) siRNA screening for top 50 genes with the highest integrative scores and upregulated in tumors. The genes that affect cell proliferation are determined by a 10% decrease or a 10% increase in cell proliferation rate in at least half of the repeated experiments after siRNA knockout.

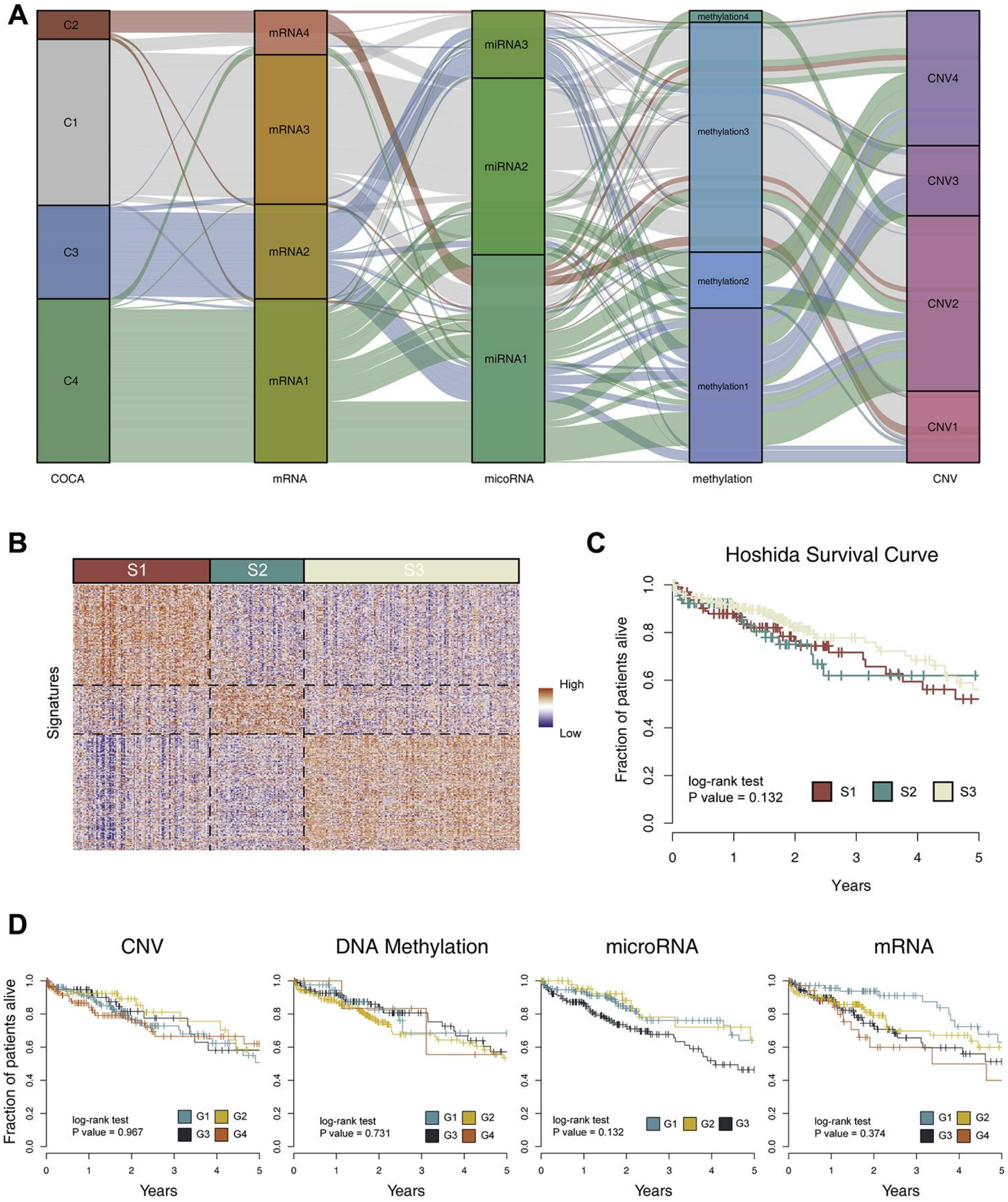

Supplementary Figure 4. Comparison of integrative classification and other single-platform methods. (**A**) The consistency of integrative clustering and single-platform clustering. (**B**) The heat-map depicts gene expression profiles of 619 genes in Hoshida's subtypes. (**C**, **D**). Kaplan–Meier estimates of the overall survival. Patients were classified according to Hoshida's signature genes or single-platform drivers.

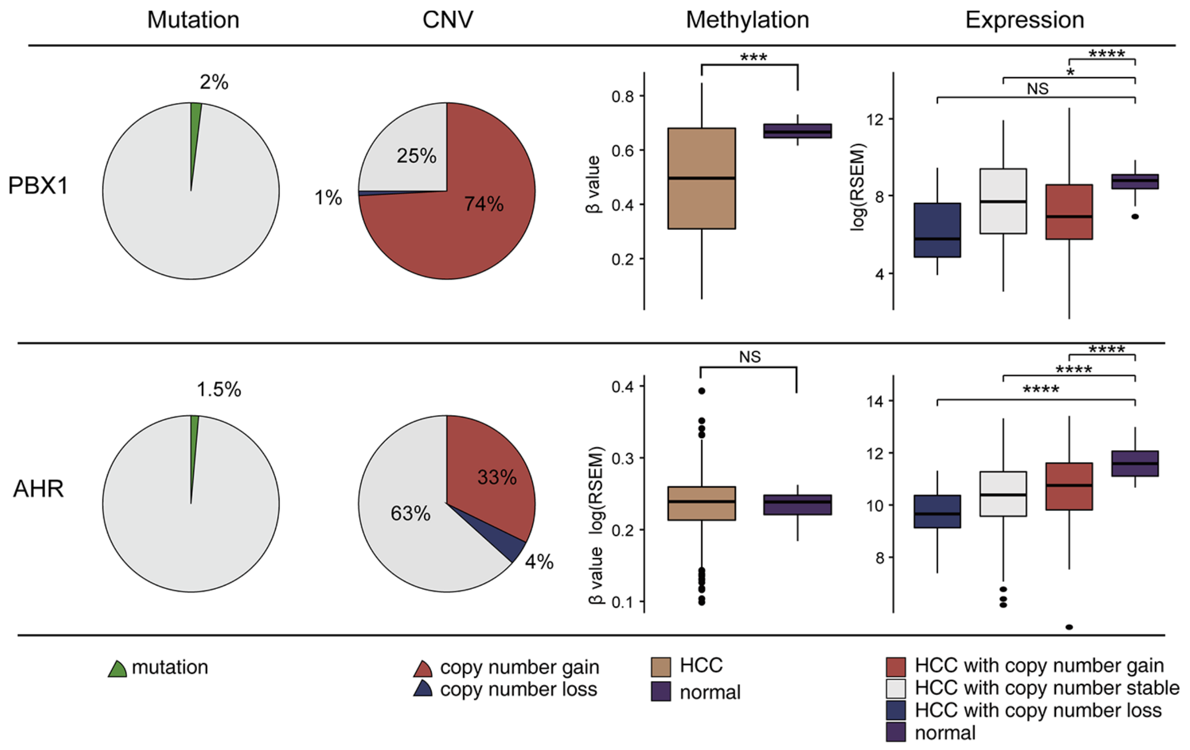

Supplementary Figure 5. Genomic alteration and expression of PBX1 and AHR. P value was determined by Wilcoxon rank sum test: ***P<0.001; **P<0.01; *P<0.05; NS:P≥0.05.

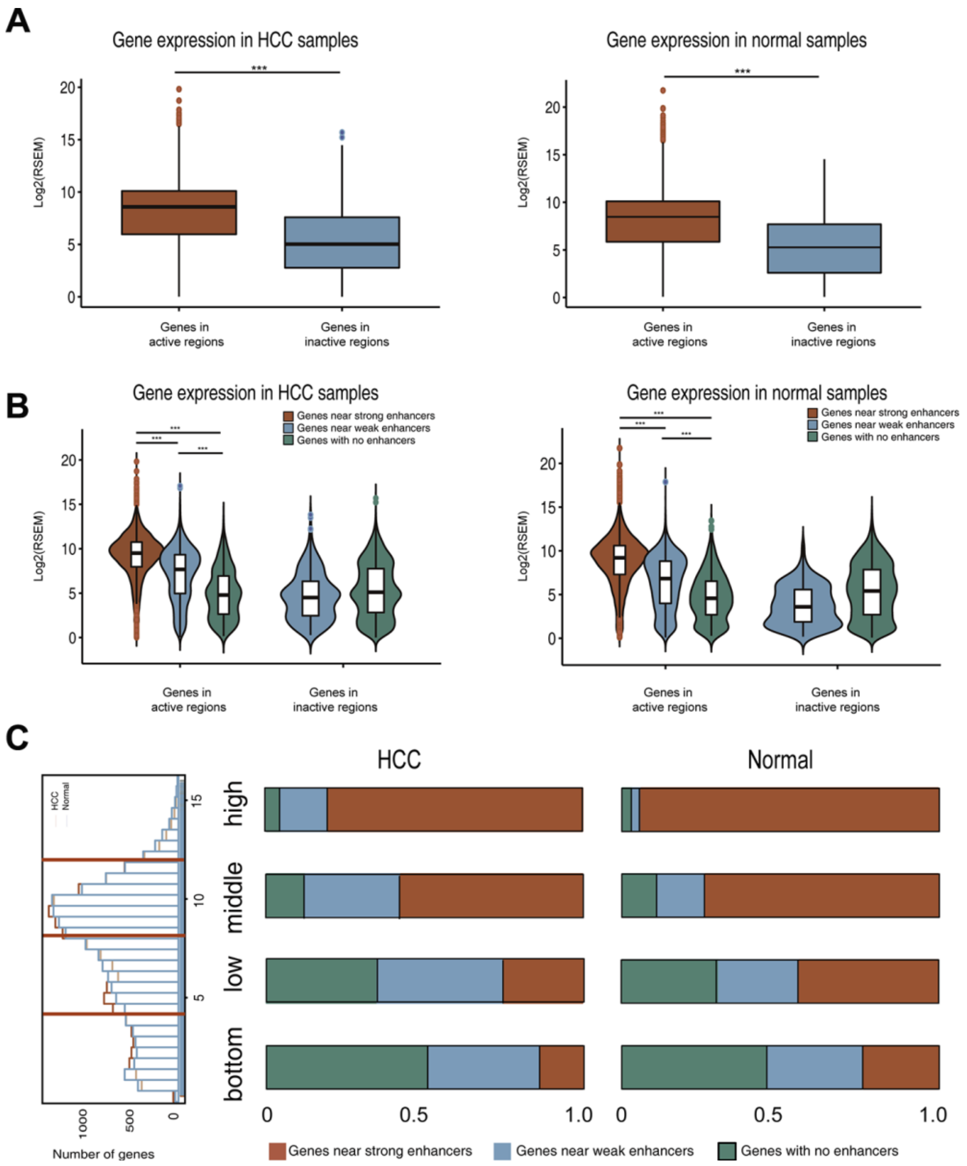

Supplementary Figure 6. Relationship of chromatin states and enhancers with gene expression in HCC and normal samples. (**A**) Genes in activation regions (chromatin states E1-E12) show higher levels of gene expression than in inactivated regions (chromatin states E13-E18). (**B**) Enhancers significantly affect gene expression in activation regions. Strong enhancers are regions marked with both H3K4me1 and H3K27ac, and weak enhancers are regions only marked with H3K4me1, excluding promoters. (**C**) High-expression genes show greater association with strong enhancers. Genes associated with genome regions are assigned by 20kb from the TSS. P value was determined by Wilcoxon rank sum test: ***P < 0.001; **P < 0.01; *P < 0.05.

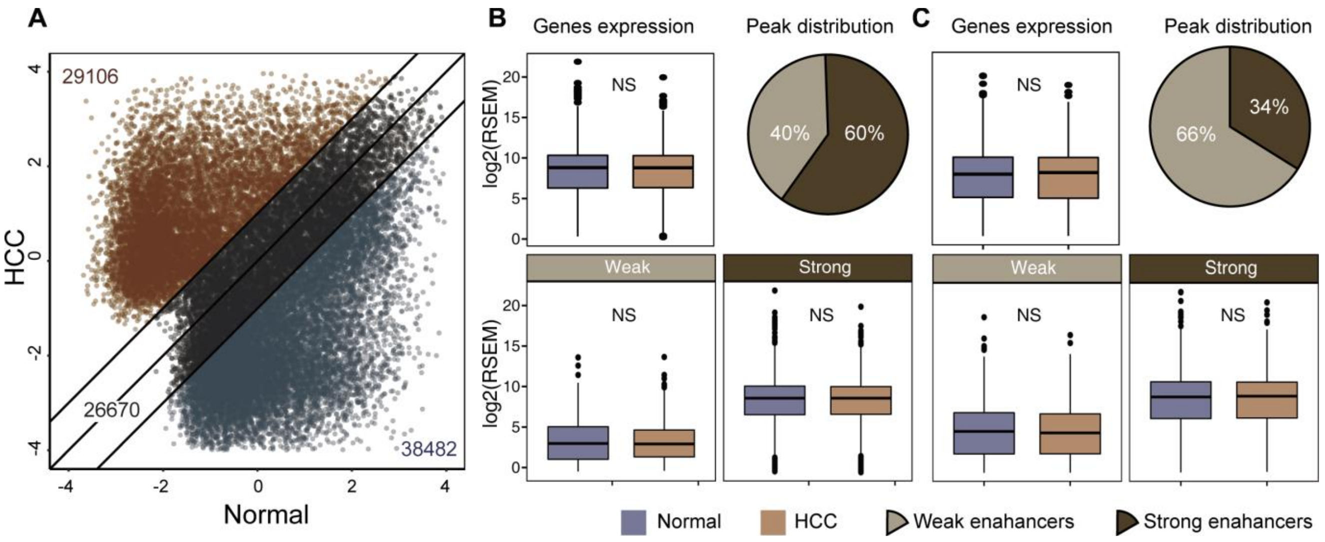

Supplementary Figure 7. Enhancer subgroups and gene expression in stable and gain regions. (**A**) Scatter plot of H3K4me1 signal in HCC cell line (HepG2) and normal liver tissue. Lost, stable, and gained peaks were labelled by blue, black, and red, respectively. (**B**, **C**) Gene expression in enhancer stable and gained regions. P value was determined by student's t test: ***P<0.001; **P<0.01:*P<0.05; NS P≥0.05.
